# Investigations Relating to the Etiology and Prophylaxis of Yellow Fever

**Published:** 1888-06

**Authors:** George M. Sternberg

**Affiliations:** Major and Surgeon U. S. Army


### Selections.

### INVESTIGATIONS RELATING TO THE ETIOLOGY AND PROPHYLAXIS OF YELLOW FEVER.*

BY GEORGE M. STERNBERG, M. D., MAJOR AND SURGEON U. S. ARMY.

GENTLEMEN—You are aware that I have been engaged during the past year in an investigation of the methods of inoculation practiced in Brazil and in Mexico, by which it is claimed that protection against yellow fever is afforded. The subject is one of such great interest to the medical profession everywhere, and of such importance with reference to the sanitary interests of that portion of the United States subject to invasion by the disease in question, that Congress provided for an investigation of the methods of inoculation practiced in Brazil and in Mexico, in the Act providing for the civil expenses of the government for the year ending June 30, 1888.

Having been selected by the President to make the investigation referred to, in compliance with my instructions I first proceeded to Brazil for the purpose of investigating the methods of Dr. Domingos Friere, of Rio de Janeiro, and after my return from that country went to Mexico to make a similar research with reference to the value of the method of inoculation practiced by Dr. Carmona y Valle. My detailed report was submitted to the President about a month ago, and by his permission I am now authorized to make public the conclusions reached. As I am about to go to Havana for the purpose of continuing my researches with reference to the etiology and prophylaxis of

* An extract from an address delivered before the College of Physicians, of Philadelphia, April 21, 1888. From the *Medical News*.

yellow fever, I am glad to avail myself of the kind invitation of the College of Physicians, of Philadelphia, and to present to you, and to the profession generally, the conclusions which I have reached up to the present date.

These conclusions are given as follows in my report above referred to:

"Facts relating to the endemic and epidemic prevalence of yellow fever, considered in connection with the present state of knowledge concerning the etiology of other infectious diseases, justify the belief that yellow fever is due to a living micro-örganism capable of development, under favorable local and meteorological conditions external to the human body, and of establishing new centres of infection when transported to distant localities.

"Inasmuch as a single attack of yellow fever, however mild, protects, as a rule, from future attacks, there is reason to hope that similar protection would result if a method could be discovered of inducing a mild attack of the disease by inoculation or otherwise.

"The hypothetical yellow fever germ, multiplying external to the human body in unsanitary places in tropical regions where the disease is endemic, or during the summer months in the area of its occasional epidemic prevalence, establishes infected localities, and susceptible persons contract yellow fever by exposure in these infected areas. We infer, therefore, *a priori*, that the yellow fever germ invades the system by the respiratory tract, by the alimentary canal, or from the general surface of the body, and it should be found in the blood and tissues, or in the alimentary canal, or upon the surface.

"Another possibility presents itself, viz., that the germ, multiplying in insanitary localities external to the body, produces a volatile poison which contaminates the air, and that an attack is induced by the toxic effects of this potent chemical poison. The more or less prolonged period of incubation—two to five days in numerous cases in which the attack has been developed after removal from the infected locality—seems opposed to this latter hypothesis.

"In the light of what is known of the etiology of other infectious diseases, the hypothesis that the germ really finds entrance to the body of the person attacked and multiplies within it, is that which presents itself as most probable, and it hardly seems worth while to consider any other unless this is proved by a complete investigation not to be true. In the latter event, we would have to consider the possibility of absorption through the respiratory tract of a volatile toxic agent, or through the skin of a poisonous ptomaine formed upon the surface of the body by a specific microörganism which does not itself penetrate to the interior.

"Naturally the attention of investigators has first been given to a search for the 'germ' in the blood of those attacked, and in the blood and tissues of the victims of the malady.

"The researches made up to the present time have failed to demonstrate the constant presence of any microörganism in the blood and tissues of those attacked.

"My own researches, recorded in the foregoing report, show that no such microörganism as Dr. Domingos Friere, of Brazil, has described in his published works, or as he presented to me in his yellow fever germ at the time of my visit to Brazil, is found, as he asserts, in the blood and tissues of typical cases of yellow fever.

"There is no satisfactory evidence that the method of inoculation practiced by Dr. Domingos Friere has any prophylactic value.

"The claims of Dr. Carmona y Valle, of Mexico, to have discovered the specific cause of yellow fever have likewise no scientific basis, and he has failed to demonstrate the protective value of his proposed method of prophlyaxis."

